# Catalytic Descriptors to Investigate Catalytic Power in the Reaction of Haloalkane Dehalogenase Enzyme with 1,2-Dichloroethane

**DOI:** 10.3390/ijms22115854

**Published:** 2021-05-29

**Authors:** Xin Xin, Chen Li, Delu Gao, Dunyou Wang

**Affiliations:** College of Physics and Electronics, Shandong Normal University, Jinan 250014, China; xin@stu.sdnu.edu.cn (X.X.); lichen8@stu.sdnu.edu.cn (C.L.); gaodelu@stu.sdnu.edu.cn (D.G.)

**Keywords:** enzyme catalysis, reactive descriptor, catalytic power, nucleophilic substitution reaction, reaction path, transition state, S_N_2 reaction

## Abstract

Enzymes play a fundamental role in many biological processes. We present a theoretical approach to investigate the catalytic power of the haloalkane dehalogenase reaction with 1,2-dichloroethane. By removing the three main active-site residues one by one from haloalkane dehalogenase, we found two reactive descriptors: one descriptor is the distance difference between the breaking bond and the forming bond, and the other is the charge difference between the transition state and the reactant complex. Both descriptors scale linearly with the reactive barriers, with the three-residue case having the smallest barrier and the zero-residue case having the largest. The results demonstrate that, as the number of residues increases, the catalytic power increases. The predicted free energy barriers using the two descriptors of this reaction in water are 23.1 and 24.2 kcal/mol, both larger than the ones with any residues, indicating that the water solvent hinders the reactivity. Both predicted barrier heights agree well with the calculated one at 25.2 kcal/mol using a quantum mechanics and molecular dynamics approach, and also agree well with the experimental result at 26.0 kcal/mol. This study shows that reactive descriptors can also be used to describe and predict the catalytic performance for enzyme catalysis.

## 1. Introduction

Enzyme catalysis not only plays an essential role in life processes [1,2], but also is of crucial importance in environmental detoxification processes [3]. Thus, to understand the catalytic power of enzymes on an atomic level is fundamentally important. In general, there have been many proposals to elucidate the catalytic power of enzymes [4,5,6]. The two most common explanations are electrostatic effects [4,7,8,9,10] and dynamical effects [11,12,13,14,15,16,17]. The electrostatic effects’ explanations emphasize that the most important catalytic factor is the stabilization of the transition state by electrostatic preorganization of the enzyme active site and that other effects usually are relatively small. The dynamical effects’ explanations state that the enzyme plays a role to optimize a particular vibrational mode for moving the system to the transition state, or for converting a system at the transition state to the product state. However, there has been no consensus on the catalytic effects of enzymes.

In environmental detoxification, the enzyme haloalkane dehalogenase (DhlA), isolated from bacterial enzyme Xanthobacter autotrophicus GJ10 [3], is involved in degrading a major pollutant 1,2-dichloroethane (DCE). In recent years, its substrate specificity [18], genetics [19,20], structure [21,22] and catalytic mechanism have been extensively studied. Nonetheless, the origin of the enzyme power is still ambiguous [6]. The first step of its reaction mechanism is a nucleophilic substitution reaction (S_N_2) as the side chain aspartic acid residue Asp124 ($\mathrm{Asp}-CO_{2}^{-}$) in the DhlA attacks the DCE [23,24,25,26,27,28] to catalyze the displacement of *Cl*^−^. Franken et al. [21] determined the three-dimensional structure of DhlA and suggested that Asp124 is the nucleophile for the catalysis. Verschueren et al. [29] showed that catalysis by Dh1A is a two-step mechanism. The X-ray structure study by Schanstra et al. [23] proposed that, besides the residues of Trp125 and Trp175 in DhlA, Phe172 is also involved in stabilizing the activate site of the enzyme. Molecular modeling methods were carried out by Dambordky et al. [24,25] to investigate the dehalogenase reaction mechanism and to determine the rate-limiting step. Theoretical simulations based on empirical valence bond potential were performed to study the catalytic effect of Dh1A [26] and found that the electrostatic effects are responsible for most of the catalytic effects. Several versions of quantum mechanics and molecular mechanics (QM/MM) [27,28] as well as molecular dynamics (MD) [28] simulations were carried out to study the kinetic isotope effects [27], activation barriers [27,28,30], and comparison with the corresponding reaction in water [4,5,6,13,27,28,30,31].

**Figure 1:**
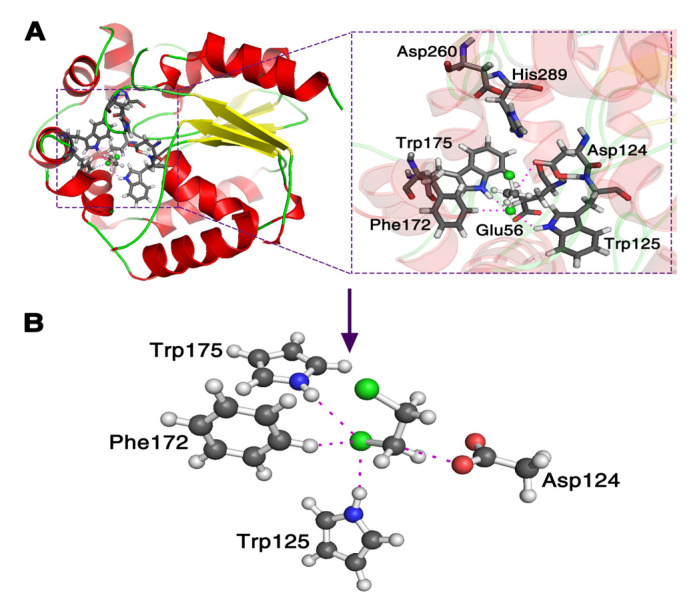
Theoretical model of the enzyme haloalkane dehalogenase (DhlA) catalytic reaction with 1,2-dichloroethane (PDB code 2DHC in (**A**)). The side chain Asp124 ($\mathrm{Asp}-CO_{2}^{-}$) in the DhlA is replaced with $CH_{3}CO_{2}^{-}$ and three residues of Trp125, Trp175 and Phe172 are reduced using their active sites, respectively, in (**B**).

It is still computationally formidable to simulate the whole system at the pure quantum mechanics level. Thus, first, we replace the nucleophile $\mathrm{Asp}-CO_{2}^{-}$ with $CH_{3}CO_{2}^{-}$ ($AcO^{-}$) due to the fact they have the same active site; in addition, we simplify the three residues Trp125, Trp175 and Phe172 using their active sites to represent them as seen in Figure 1. Second, we take away the three residues one by one to investigate the reactive descriptors, to explain the catalytic power of enzyme DhlA in the S_N_2 step. Third, using the reactive descriptors, the reactive barrier of the corresponding *AcO*^−^ attacking DCE reaction in a water environment is predicted, and compared with the one calculated using a quantum mechanics and molecular mechanics (QM/MM) method [32,33].

Reactive descriptors have been established, for the past two decades, to describe and predict the catalytic reaction on heterogeneous catalysis on surfaces [34]. Until now, the reactive descriptors studied have been mostly limited to metal, metal-oxide or other surfaces [35]. Here, the purpose of this paper is to extend the studies on the reactive descriptors to explain the catalytic power in enzyme catalysis. We present a theoretical study, for the first time using reactive descriptors, to investigate the catalytic power of Asp124 catalyzing DCE in the S_N_2 step. Moreover, we use the obtained descriptors to predict the performance of a water environment in this S_N_2 reaction, and use the obtained relationships between reactive descriptors and the activation barriers to predict the barrier of this S_N_2 reaction in water.

## 2. Results and Discussion

### 2.1. The Bond Breaking and Formation Descriptor

**Figure 2:**
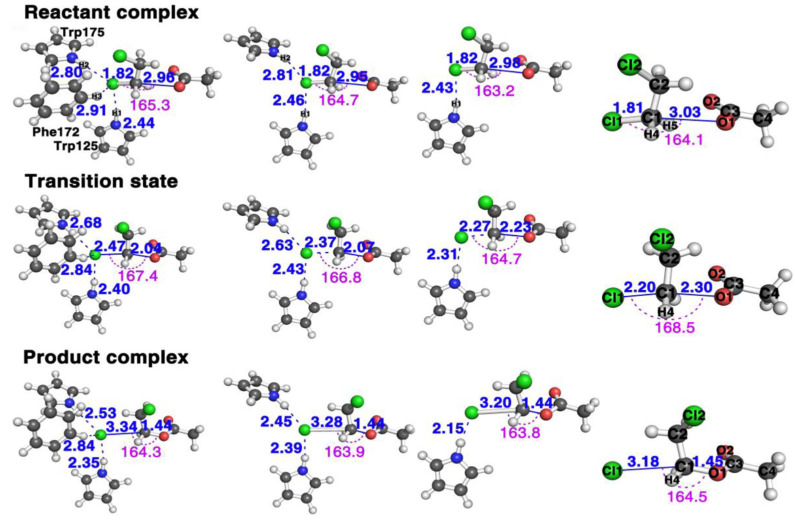
The structural comparison of the three stationary points, reactants (top row), transition states (middle row), and products (bottom row) of *AcO*^−^ + *DCE* reaction in the presence of, from left column to right column, three (Trp125 + Phe172 + Trp175), two (Trp125 + Trp175), one (Trp125) and zero residues, respectively (see Figures S1–S5 for more detailed information of these geometries in the Supplementary Materials).

Figure 2 shows the structures of the stationary points along the reaction pathways of the reactions with three (Trp125 + Phe172 + Trp175), two (Trp125 + Trp175), one (Trp125), and zero residues, respectively.

**Figure 3:**
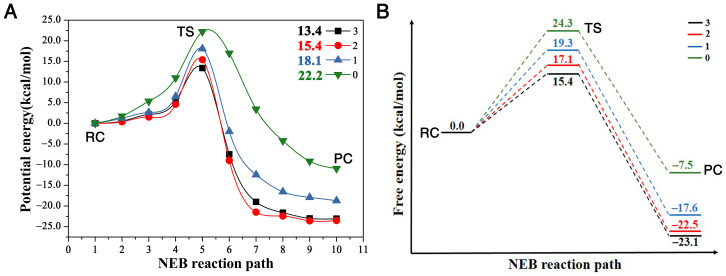
(**A**) is the potential energy of $AcO^{-}+DCE$ reaction in the presence of three (Trp125 + Phe172 + Trp175), two (Trp125 + Trp175), one (Trp125), and zero residues, along the NEB reaction path, respectively; (**B**) is a schematic plot of the reaction profile showing the free energies at the stationary points along the NEB reaction path. (RC, TS and PC repreScheme 0. Å for the reaction without any residues. The order of this $D_{cat}$ descriptor with different numbers of residues is $D_{cat}\left(3\right)>D_{cat}\left(2\right)>D_{cat}\left(1\right)>D_{cat}\left(0\right)$. Therefore, this $D_{cat}$ behavior proves that the presence of the enzyme, even only with one residue, catalyzes the S_N_2 reaction of $AcO^{-}+DCE$. The reactivity order R_N_ is $R_{3}>R_{2}>R_{1}>R_{0}$ (N infers the number of residues).

This S_N_2 mechanism has the concerted formation of the O1–C1 bond and the breaking of the C1 and Cl1 leaving group. The faster the C1–Cl1 bond breaks and the O1–C1 forms, the faster it finishes the reaction, and the bigger the reactivity. In other words, we may say it has a “later” transition state. By comparing the transition state geometries, we can see which case has a faster bond breaking and bond formation process. First, let us define one parameter $D_{cat}=d_{l}-d_{f}$ as the reactivity catalytic descriptor. Here, $d_{l}$ represents the distance between the leaving group Cl1 and C1 in the substrate and $d_{f}$ the forming distance between the O1 in the nucleophile and C1 in the substrate. Thus, $D_{cat}$ represents the difference between the breaking bond and the forming bond. The bigger the $D_{cat}$ value at the transition state, the faster the Cl1–C1 bond breaks (the bigger the breaking bond distance) and the faster the O1–C1 bond forms (the shorter the forming bond distance); thus, the more “lateness” of the transition state and the larger the reactivity. More specifically, there should be a larger number of reactants passing the transition state per unit time according to the transition state theory [36], namely, the larger the turn-over frequency, which associates with a lower activation barrier. We can see that the $D_{cat}$ value is 0.43 Å for the reaction with three residues, 0.30 Å with two and only 0.04 Å with one; thus, the reaction with the all three residues (Trp125 + Phe172 + Trp175) reacts the fastest, then the reaction with two (Trp125 + Trp175), with the reaction with only one (Trp125) having the smallest reactivity. This is proven by the free energy barriers, shown in Figure 3B with three, two, one and zero residues are 15.4, 17.1, 19.3, and 24.3 kcal/mol, respectively, with the one with three residues having the smallest energy barrier and the one without any residues having the largest.

The comparison of the four reaction pathways, in Figure 3, further proves our conclusion. It shows that the reaction barriers of these four reactions have the order $∆E_{3}<∆E_{2}<∆E_{1}<∆E_{0}$, corresponding to the reactivity order $R_{3}>R_{2}>R_{1}>R_{0}$. Figure 3A shows that the potential energy barriers with three, two, one and zero residues are 13.4, 15.4, 18.1, and 22.2 kcal/mol, respectively. In order to compare our reaction barriers with the experimental and theoretical values in terms of free energy, we calculated the free energies of these stationary points with Gaussian 16 package [37], as shown in Figure 3B. The reaction free energy barrier without any residues is the largest at 24.3 kcal/mol, agreeing very well with a previous study of a MP2/6-31 + G* result at 23.2 kcal/mol [27], and the free energy barriers with one, two and three residues are 19.3, 17.1 and 15.4 kcal/mol, respectively. The free energy barrier with three residues of 15.4 kcal/mol also agrees very well with experiment result at 15.3 kcal/mol [23]. This comparison proves that the presence of the residues enhances the reactivity, i.e., catalyzes the S_N_2 reaction, compared to the case with zero residues.

**Figure 4:**
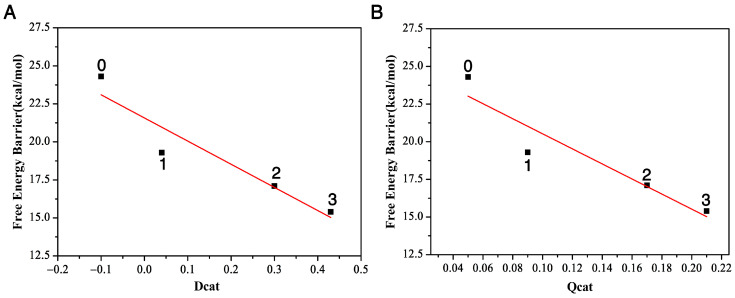
The relationship between the free energy barriers and the two descriptors, D_cat_ (**A**) and Q_cat_ (**B**), with three, two, one, and zero residues, respectively.

We plot the relationship between the distance difference descriptor $D_{cat}$ and the free energy barrier in Figure 4A. The linear relationship between these two properties describes the catalytic power and catalytic performance—that it is the surrounding residues around DCE that catalyze the S_N_2 reactivities. Additionally, the more residues that encompass the substrate, the bigger reactivity (catalytic power) the reaction has, with the three-residue case having the smallest activation barrier and largest reactivity. In other words, the presence of the enzyme speeds up the bond breaking and formation process in this S_N_2 step.

### 2.2. The Charge Transfer Descriptor

In this S_N_2 reaction process, the negative charge of nucleophile ($CH_{3}CO_{2}^{-}$) at the asymptote will gradually transfer to the leaving group Cl from the reactant complex to the transition state and then to the product complex. Let us define the charge transfer descriptor as $Q_{cat}=q_{ts}-q_{rc}$. Here, $q_{ts}$ and $q_{rc}$ are calculated as the sum of the signed atomic charge values of the nucleophile, representing the charge of the nucleophile at the transition state and reactant complex, respectively. The bigger the $Q_{cat}$ value is, the faster the charge transferred from the nucleophile to the leaving group, and the bigger the reactivity. From Table 1, we can see that this number is 0.09 with one residue Trp125, 0.17 with two residues Trp125 + Trp175 and 0.21 with three residues Trp125 + Phe172 + Trp175, and only 0.05 without any residues. The order of the charger transfer descriptor is $Q_{cat}\left(3\right)>Q_{cat}\left(2\right)>Q_{cat}\left(1\right)>Q_{cat}\left(0\right)$. This further demonstrates that the presence of the enzyme catalyzes the $AcO^{-}+DCE$ reaction. The relationship between the charge-transfer descriptor $Q_{cat}$ and the free energy barrier ∆E is plotted in Figure 4B. It also shows a linear relationship, namely that, as the number of residues increases from zero to three, the activation barrier decreases. Compared to the reaction without residues, the presence of the enzyme speeds up the charge transfer rate of the S_N_2 process, catalyzing the S_N_2 process.

So why does the presence of this enzyme enhance bond breaking and formation, as well as the charger transfer mechanism? As the S_N_2 dynamics evolves, the negative charge is gradually transferred from the nucleophile to the leaving group. As the leaving group Cl1 becomes more negative, it creates an electric field that interacts with the active-sites of the residues, giving rise to an induction energy which acts as a strong long-range attractive potential in addition to the van der Waals potential. This long-range attractive potential can significantly reduce the barrier to chemical rearrangement [38]. Additionally, this has been demonstrated in the activation barrier heights, in Figure 3A, in the presence of aromatic rings. Compared to the zero-residue case without induction energy, with the presence of the attractive induction potential energy, the barrier height with one residue Trp125 is reduced by 4.1 kcal/mol. With the two-residue case, the barrier is further lowered by another 2.7 kcal/mol since the negative Cl charge now polarizes the two residues and produces two parts of attractive induction energy. With three residues, Trp125 + Trp175 + Phe172, the barrier height is reduced further by another 2.0 kcal/mol to the smallest at 13.4 kcal/mol.

From the structural point of view in Figure 2, the three aromatic rings are all in the front side of the leaving group, i.e., the bond-breaking direction. Thus, the induction attractive potentials between the leaving group and the residues exert a pulling force from the residues to the leaving group, speeding up the bond breaking of the leaving group from the substrate, as well as the negative-charge transfer process.

### 2.3. Prediction of the Reactivity in Water

We have already obtained two descriptors of the catalytic effects in previous sections: the first descriptor is $D_{cat}$, and the second is $Q_{cat}$, both scaling linearly with the free energy barrier. Therefore, using the QM/MM approach, we can first obtain the $D_{cat}$ and $Q_{cat}$ values in water; then, according to the two descriptors, we can predict the barrier height in water to see if the water environment catalyzes or hinders the reactivity. Furthermore, the predicted barrier height can be compared with our calculated one in water using the QM/MM method to confirm the prediction values. 

**Figure 5:**
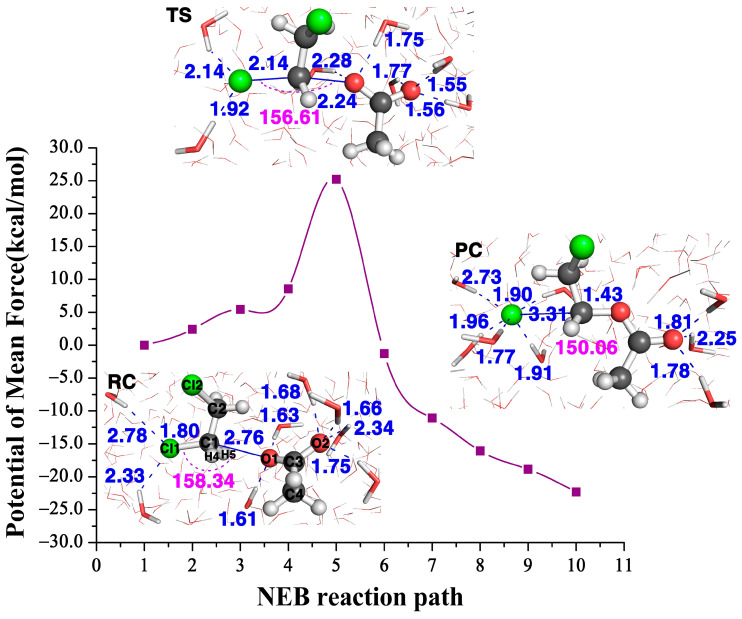
The NEB reaction path of the $AcO^{-}+DCE$ reaction and the structures of its reactant complex (RS), transition state (TS) and product complex (PC) in water.

The structures of the three stationary points (reactant complex, transition state and product complex) in the $AcO^{-}$ attacking DCE in water are inserted in Figure 5. The comparison of the $D_{cat}$ and $Q_{cat}$ values of this reaction with three residues (the far left column in Figure 2) and the ones in water tells us two different stories. At transition states, this Cl1–C1 bond length becomes 2.47 Å with three residues, while it is shorter, only 2.14 Å, in water. In the meantime, the formation bond O1–C1 is 2.04 Å with three residues, which is much longer, 2.24 Å, in water. The shorter length of the breaking bond and the longer length of the formation bond in water indicate that the water environment hinders the reactivity. Indeed, the $D_{cat}$ value is 0.43 for the reaction with three residues and is only −0.10 in water. According to the relationship between $D_{cat}$ and the free energy barrier in Figure 4A, we can predict that the reaction barrier in the water environment is about 23.1 kcal/mol, about 7.7 kcal/mol larger than with three residues at 15.4 kcal/mol. Furthermore, Table 1 tells us the $Q_{cat}$ value in water is 0.04, which is much smaller than with three residues, 0.21, in water; thus, according to the relationship between $Q_{cat}$ and the free energy barrier in Figure 4B, we can predict that the energy barrier in the water environment is about 24.2 kcal/mol, which is 8.8 kcal/mol larger than the one with three residues. Therefore, the enzyme environment enhances the reaction reactivity, while the water environment actually hinders the reactivity.

Figure 5 shows the NEB reaction pathway in water calculated with the QM/MM approach. The calculated free energy barrier height of the transition state is 25.2 kcal/mol, which agrees very well with the one predicted with the $D_{\mathrm{cat}}$ descriptor at about 23.1 kcal/mol, while also in agreement with the $Q_{\mathrm{cat}}$ predicted one at about 24.2 kcal/mol. Therefore, in contrast to the barrier height with three residues at 15.4 kcal/mol, it tells us the water solvent impedes the reactivity. Note both predicted barrier heights of 23.1 kcal/mol and 24.2 kcal/mol agree well with the calculated free energy barrier height of 25.2 kcal/mol in water, and also agree well with the experimental value at ~26.0 kcal/mol [26].

As this reaction takes place in water, on one hand, as the negative charge gradually transfers from the nucleophile $AcO^{-}$ to the leaving group Cl1, it should also induce the attractive long-range potential between the leaving group Cl1 and its surrounding water molecules. However, the water molecules surrounding the leaving group are not only in its frontside, bond-breaking direction, but also in the backside direction. Namely, the water molecules are not orderly distributed around the leaving group. As a result, the polarized water molecules do not exert a uniform, forward pulling force to the leaving group. Of course, to explicitly investigate the role of the water molecules surrounding the leaving group and the nucleophile $AcO^{-}$ in the QM/MM calculation, it would be better to include at least the water molecules in the first-solvation shell in the QM region. Nonetheless, the water molecules usually undergo of the order of 10^13^ collisions per second with the solute molecule [38], which hinders the separation between the leaving group and the substrate in the S_N_2 step. Furthermore, previous calculations using the current QM/MM method with an explicit SPC/E water model on the S_N_2 reactions of OH^−^ + CH_3_Br [39] and CN^−^ + CH_3_Cl [40], without the water molecules in the QM region, give accurate activation barriers compared to the experimental ones. 

On the other hand, at the transition state structure, O1 in the nucleophile forms two hydrogen bonds with surrounding water molecules, which reduces its negativity, thus reducing its interaction with the Cl1 center. In addition, O2 has three hydrogen bond interactions with the water molecules at its backside, pulling it back from the attacking direction. Therefore, we can conclude, in a water environment, the interactions with the surrounding water molecules, especially the hydrogen bond interactions surrounding the nucleophile, hinder its reactivity. Therefore, in a water environment, the reactivity is impeded.

## 3. Methodologies

### 3.1. Quantum Mechanics Calculation for the AcO^−^ + DCE with Three, Two, One, and Zero Residues

The reaction pathway of $AcO^{-}+DCE$ was computed using Density Function Theory (DFT) quantum level of theory with 6-31 + G** basis set [41]. The M08-HX exchange correlation functional was used for the DFT calculation due to its accuracy in predicting the bond lengths of aromatic rings, the non-covalent interactions of larger molecules and the reaction barrier heights [42]. First, the structure in Figure 1B was optimized as the reactant complex, and the product complex was searched from the reaction complex according to the S_N_2 mechanism. Second, the Nudged Elastic Band (NEB) method [43] was adopted to construct the reaction pathway using the reactant complex and product complex. Then, the top structure on the reaction pathway was confirmed as the transition state with one imaginary vibrational frequency. The displacements of the imaginary-vibrational-frequency mode of the transition state were used to locate the final reactant and product complexes. Finally, the final NEB reaction pathway was constructed based on the final reactant, transition state and product complexes. The evolutions of the structures, charges, and energies along the reaction pathway of the whole reaction system were then determined. 

In this theoretical study, the Trp125, Trp175, and Phe172 residues were removed one by one from the enzyme to see the partial and whole enzyme catalytic effects on the reaction pathways. The calculations of the reaction pathways of $AcO^{-}+DCE$ with two aromatic rings (Trp125, Trp175), with one aromatic ring (Trp 125) and without aromatic rings were carried out using the same method, respectively.

### 3.2. Quantum Mechanics and Molecular Mechanics Calculation for AcO^−^ + DCE in Water

For the $AcO^{-}+DCE$ reaction in water, we employed a QM/MM approach [32,33] to calculate its reaction path in water. We treated the whole reaction system as two parts: we used the quantum mechanical method to treat the solute region $AcO^{-}+DCE$ and molecular mechanics method for the water molecules. The QM solute was solvated into a 37.5 Å cubic water box with 1762 water molecules described by an explicit SPC/E water model [44]. The potential energy of the whole system can be expressed as:

(1) $$V=V_{qm}(r,\varphi )+V_{qm/mm}(r,R,\rho )+V_{mm}(R)$$

The first term $V_{qm}\left(r,\varphi \right)$ represents the QM energy of the solute subsystem in the gas phase. The r and φ in the expression represent the coordinates and ground-state electronic wave function of the QM region, respectively. The second term $V_{qm/mm}\left(r,R,\rho \right)$ describes the solute and solvent electrostatic interactions. The solute–solvent bond interactions, and van der Waals interactions, as well as the interactions of the solvent subsystem are included in the third term $V_{mm}\left(R\right)$, where R stands for the coordinate of the MM region. The electrostatic interaction contribution can be described approximately by effective electrostatic potential (ESP) for the solute–solvent interactions [45]. The ESP representation forms the base-level theory for the QM region. For the DFT level of theory calculation, the same DFT/M08-HX/6-31 + G** combination is used as described in Section 2.1.

The potential of mean force (PMF) along the reaction pathway in water solution was calculated as:

(2) $$W(r,\beta )=-\frac{1}{\beta }\ln\int e^{-\beta V(r,R;\varphi )}dR\;\;\;\beta =\frac{1}{kT}$$

After the $AcO^{-}+DCE$ reactant complex was solvated and optimized using a multi-region optimization in water, we searched the product complex according to the S_N_2 mechanism, and then constructed an initial NEB reaction pathway. In this initial NEB reaction pathway, we confirmed the top structure on the reaction pathway with one imaginary frequency. Based on this transition state, along the negative frequency vibrational mode, we searched and determined the new reactant and product complexes to construct the NEB pathway again. The dynamical equilibration of the solvent water molecules for 120 ps and then the optimization of the entire reaction pathway were performed until the reaction pathway converged. The above reaction pathway calculations were performed using the NWChem package [46].

## 4. Conclusions

In this paper, we present a theoretical study of using reactive descriptors to explore the catalytic power in the S_N_2 reaction of the haloalkane dehalogenase enzyme with 1,2-dichloroethane. We found two catalytic descriptors that have a linear relationship with the reaction barriers. One is the distance difference $D_{cat}$ of the breaking bond and the forming bond, and the other is the charge difference $Q_{cat}$ of the nucleophile between the transition state and reactant complex. Both descriptors demonstrate that as the number of residues increases, the reaction barriers decrease, thus the catalytic power increases. The two descriptors indicate that the presence of the residues speeds up the S_N_2 bond breaking and formation process and also enhances the charge transfer process. Therefore, the presence of enzymes catalyzes the S_N_2 reaction.

The two catalytic descriptors were also used to predict the reaction barrier of this reaction in water. The activation barrier predicted with the $D_{cat}$ descriptor is about 23.1 kcal/mol, and the one predicted with $Q_{cat}$ is about 24.2 kcal/mol. Both agree well with the experimental result at 26.0 kcal/mol. These results mean that the water environment hinders this S_N_2 reaction. The two descriptors we found in this theoretical study show that reactive descriptors can also be developed in enzyme catalysis to explain the catalytic power and to describe the performance of its reactivity. They might also be used to design effective enzyme catalytic reactions such as in environmental detoxification processes.

## Figures and Tables

**Table 1:** The charge distributions of the key atoms or groups of the reactant complexes (RC), transition states (TS) for the $AcO^{-}+DCE$ reaction in the presence of three, two, one and zero residues, as well as the reaction in water solution. (The detailed charge distributions of the RC, TS and product complexes are listed in Table S1 of the Supplementary Materials.)

	3		2		1		0		Water	
	RC_3_	TS_3_	RC_2_	TS_2_	RC_1_	TS_1_	RC_0_	TS_0_	RC_sol_	TS_sol_
O1	−0.88	−0.80	−0.87	−0.81	−0.87	−0.86	−0.86	−0.85	−0.80	−0.55
C_2_H_4_Cl	0.33	0.53	0.34	0.52	0.33	0.52	0.31	0.47	0.27	0.42
Cl1	−0.34	−0.70	−0.36	−0.68	−0.35	−0.63	−0.37	−0.58	−0.28	−0.46
H1	0.26	0.27	0.28	0.29	0.27	0.32				
H2	0.30	0.31	0.29	0.31						
H3	0.11	0.09								
CH_3_CO_2_	−0.94	−0.73	−0.94	−0.77	−0.94	−0.85	−0.94	−0.89	−0.66	−0.62

## Data Availability

The data presented in this study are available in this article and supplementary materials.

## Supplementary Materials

The Supplementary Materials are available online at https://www.mdpi.com/article/10.3390/ijms22115854/s1.
