# Supplementary material for: Catalytic Descriptors to Investigate Catalytic Power in the Reaction of Haloalkane Dehalogenase Enzyme with 1,2-Dichloroethane
Source: Int J Mol Sci. 2021 May 29;22(11):5854. doi: 10.3390/ijms22115854 (PMC8197811; doi:10.3390/ijms22115854)
Supplement: Supplementary file 1 [file ijms-22-05854-s001.zip › ijms-1214204-supplementary.pdf]

Supplementary Materials

Catalytic Descriptors to Investigate Catalytic Power in the Reaction of Haloalkane Dehalogenase Enzyme with 1,2-dichloroethane

Xin Xin, Chen Li, Delu Gao and Dunyou Wang*

College of Physics and Electronics, Shandong Normal University, Jinan Shandong 250014, China

* Correspondence: dywang@sdnu.edu.cn

Table S1. The charge distributions of the reactant complexes (RC), transition states (TS), and product complexes (RC) for the AcO⁻+DCE in the presents of three, two, one and zero residues, as well as the reaction in water solution.

3	RC3	TS3	PC3	2	RC2	TS2	PC2
C1	0.319033	0.574075	0.669512	C1	0.274441	0.538365	0.685243
H5	0.073417	0.018371	-0.071114	H5	0.089538	0.029579	-0.069969
H4	-0.015057	0.009279	-0.044495	H4	-0.006499	0.017421	-0.047489
C2	0.248677	0.130487	-0.011599	C2	0.286908	0.176117	0.017404
H	0.022054	0.048066	0.096569	H	0.014341	0.033427	0.072154
H	0.076923	0.070507	0.068384	H	0.071772	0.060598	0.063162
Cl2	-0.390375	-0.320147	-0.299771	Cl2	-0.394744	-0.334762	-0.317333
Cl1	-0.340244	-0.696101	-0.831223	Cl1	-0.357263	-0.682120	-0.833798
C4	-0.290802	-0.323744	-0.366941	C4	-0.291037	-0.331233	-0.359889
C3	1.018606	1.017157	0.935062	C3	1.005898	1.022750	0.934793
O2	-0.875430	-0.791477	-0.664432	O2	-0.874664	-0.810334	-0.669016
O1	-0.875732	-0.803198	-0.634971	O1	-0.867769	-0.811846	-0.640192
C	-0.219535	-0.212574	-0.208892	C	-0.200087	-0.202013	-0.200036
C	-0.166929	-0.156523	-0.134186	C	-0.181976	-0.167833	-0.141281
C	-0.129186	-0.156632	-0.171632	C	-0.146599	-0.161496	-0.175634
N	-0.078338	-0.083566	-0.131615	N	-0.096304	-0.103158	-0.152100
C	-0.222534	-0.209118	-0.188503	C	-0.200886	-0.198285	-0.174338
C	-0.081927	-0.124462	-0.104861	C	-0.240413	-0.230963	-0.228734
C	-0.115089	-0.050353	-0.112617	C	-0.138199	-0.137674	-0.073400
C	-0.089732	-0.084321	-0.114301	C	-0.128465	-0.138109	-0.152670
C	-0.123110	-0.222113	-0.039691	N	-0.010905	-0.087393	-0.237981
C	-0.117120	-0.144348	-0.105285	C	-0.255007	-0.213752	-0.142062
C	-0.077355	0.017012	-0.114442	H	0.100121	0.098013	0.093027
C	-0.189300	-0.167000	-0.155746	H	0.108766	0.104069	0.097205
C	-0.174854	-0.198519	-0.182364	H	0.146261	0.140039	0.131704
C	-0.128091	-0.144247	-0.177201	H1	0.276652	0.292104	0.334639
N	0.007592	-0.051569	-0.099250	H	0.153358	0.144439	0.131489
C	-0.355864	-0.314955	-0.258538	H	0.025470	0.053123	0.071439
H	0.096856	0.095736	0.093565	H	0.025660	0.056217	0.101527
H	0.112378	0.105658	0.100577	H	0.033763	0.056206	0.090735
H	0.148430	0.138566	0.127864	H2	0.288404	0.311159	0.381109
H1	0.258683	0.272320	0.325522	H	0.139235	0.132705	0.112672
H	0.158396	0.144200	0.132504	H	0.095347	0.092227	0.084913
H	0.023118	0.054086	0.073703	H	0.118557	0.111375	0.101641
H	0.024391	0.057898	0.101932	H	0.136326	0.141038	0.111067
H	0.033689	0.059766	0.093743				
H3	0.109412	0.090418	0.139772				
H	0.100077	0.106737	0.101928				
H	0.080190	0.079820	0.082589				
H	0.081121	0.067026	0.080245				

H	0.146082	0.160611	0.063764				
H	0.075542	0.081317	0.079642				
H2	0.295825	0.308655	0.348985				
H	0.227073	0.223919	0.203502				
H	0.088208	0.085530	0.090421				
H	0.099407	0.090230	0.086244				
H	0.131422	0.147518	0.127642				
1	RC1	TS1	PC1	0	RC0	TS0	PC0
C1	0.298387	0.531546	0.696248	C1	0.285914	0.407906	0.688877
H5	0.072791	0.028515	-0.063385	H5	0.057669	0.058832	-0.061540
H4	-0.005222	0.016163	-0.035397	H4	-0.003493	0.032321	-0.033321
C2	0.247668	0.140626	0.014377	C2	0.282811	0.268501	0.070245
H	0.016477	0.042651	0.113898	H	0.007730	0.003330	0.095912
H	0.069099	0.083150	0.053697	H	0.055321	0.049276	0.034587
Cl2	-0.368556	-0.321946	-0.326243	Cl2	-0.375289	-0.349032	-0.342130
Cl1	-0.347673	-0.625741	-0.901436	Cl1	-0.371353	-0.581929	-0.945080
C4	-0.318767	-0.357366	-0.354967	C4	-0.335687	-0.425527	-0.344428
C3	1.020860	1.050108	0.940783	C3	1.006758	1.026952	0.936813
O2	-0.874753	-0.845391	-0.672491	O2	-0.859288	-0.847475	-0.673046
O1	-0.870232	-0.856097	-0.662547	O1	-0.864204	-0.845464	-0.672436
C	-0.214694	-0.221349	-0.190129	H	0.034611	0.070218	0.082970
C	-0.187562	-0.156654	-0.165172	H	0.039062	0.062917	0.062063
C	-0.134258	-0.146812	-0.202778	H	0.039438	0.069174	0.100515
N	-0.074627	-0.122574	-0.146039				
C	-0.214871	-0.196040	-0.156259				
H	0.098426	0.096023	0.094575				
H	0.112254	0.108343	0.090514				
H	0.152344	0.135528	0.121956				
H1	0.265772	0.317595	0.365437				
H	0.156259	0.142309	0.126825				
H	0.030492	0.050986	0.068303				
H	0.032121	0.053996	0.103026				
H	0.038264	0.052431	0.087202				
solution	RCsol	TSsol	PCsol				
C1	0.288308	0.326662	0.303541				
H5	0.017533	0.054385	0.094925				
H4	0.052767	0.076448	0.000875				
C2	-0.010694	-0.025683	0.024184				
H	0.073048	0.112556	0.131848				
H	0.133903	0.143368	0.088592				
Cl2	-0.282930	-0.267771	-0.224736				
Cl1	-0.280540	-0.462752	-0.786197				
C4	-0.432831	-0.301531	-0.528092				
C3	0.886653	0.716257	1.063384				

O2	-0.680113	-0.737210	-0.783597				
O1	-0.795450	-0.546194	-0.594058				
H	0.098008	0.097513	0.159525				
H	0.140337	0.074445	0.146364				
H	0.122794	0.077759	0.147246				

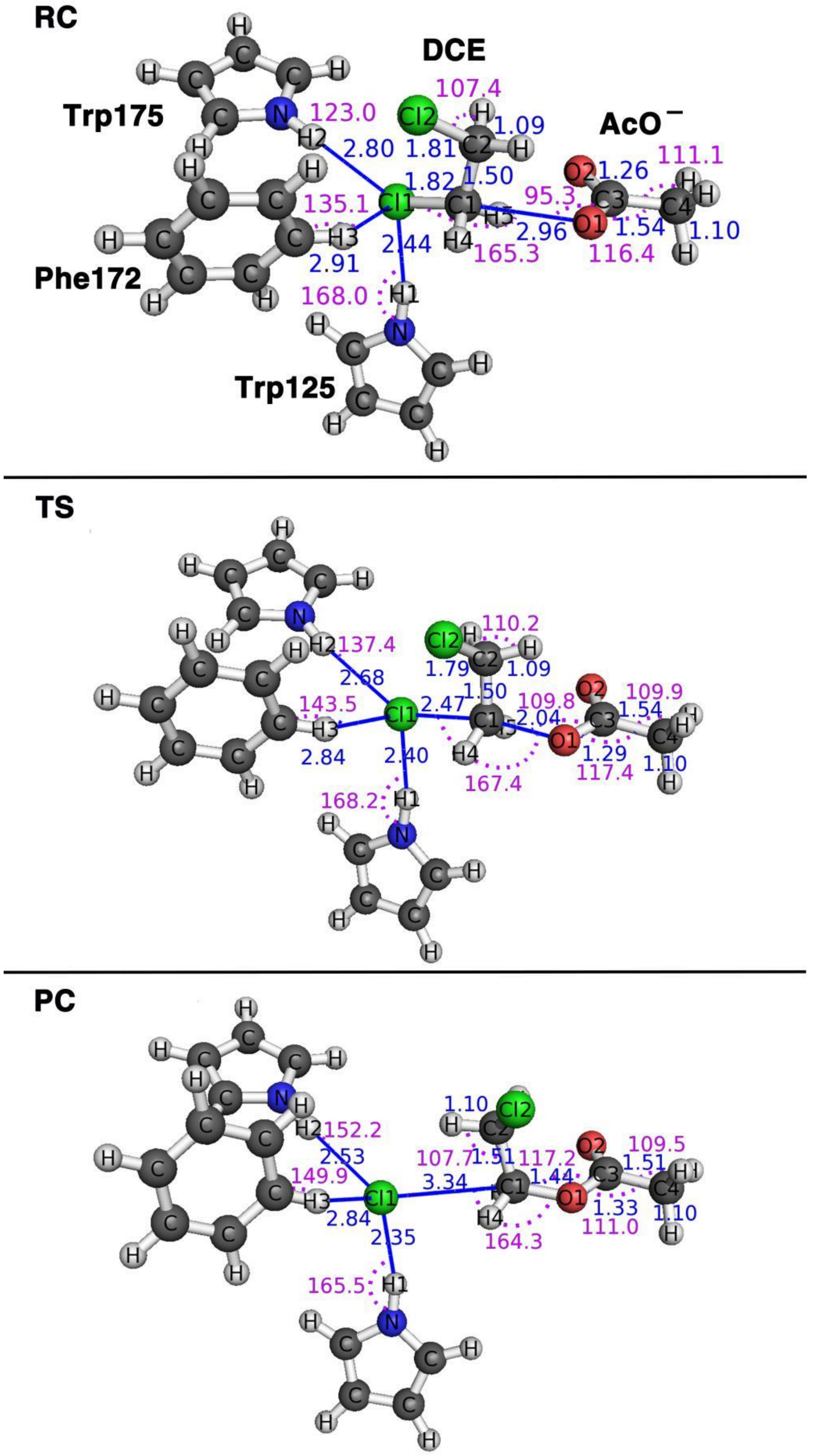

Figure S1. The three stationary points (reactant complex (RC), transition state (TS), and product complex (RC)) of the AcO^{-} + DCE reaction with three residues (Trp125+Phe172+Trp175)

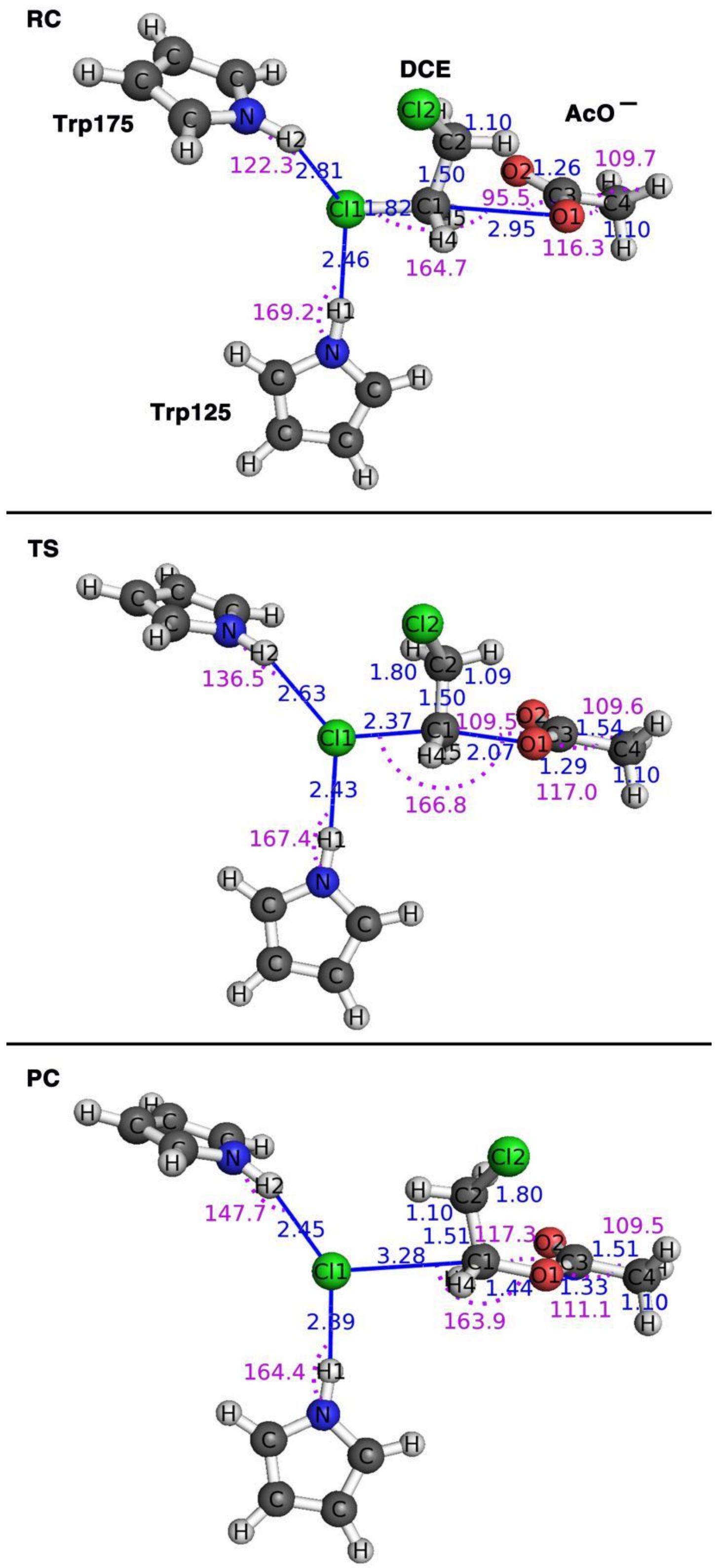

Figure S2. The three stationary points (reactant complex(RC), transition state(TS), and product complex(RC)) of the AcO^{-} + DCE reaction with two residues (Trp125+Trp175).

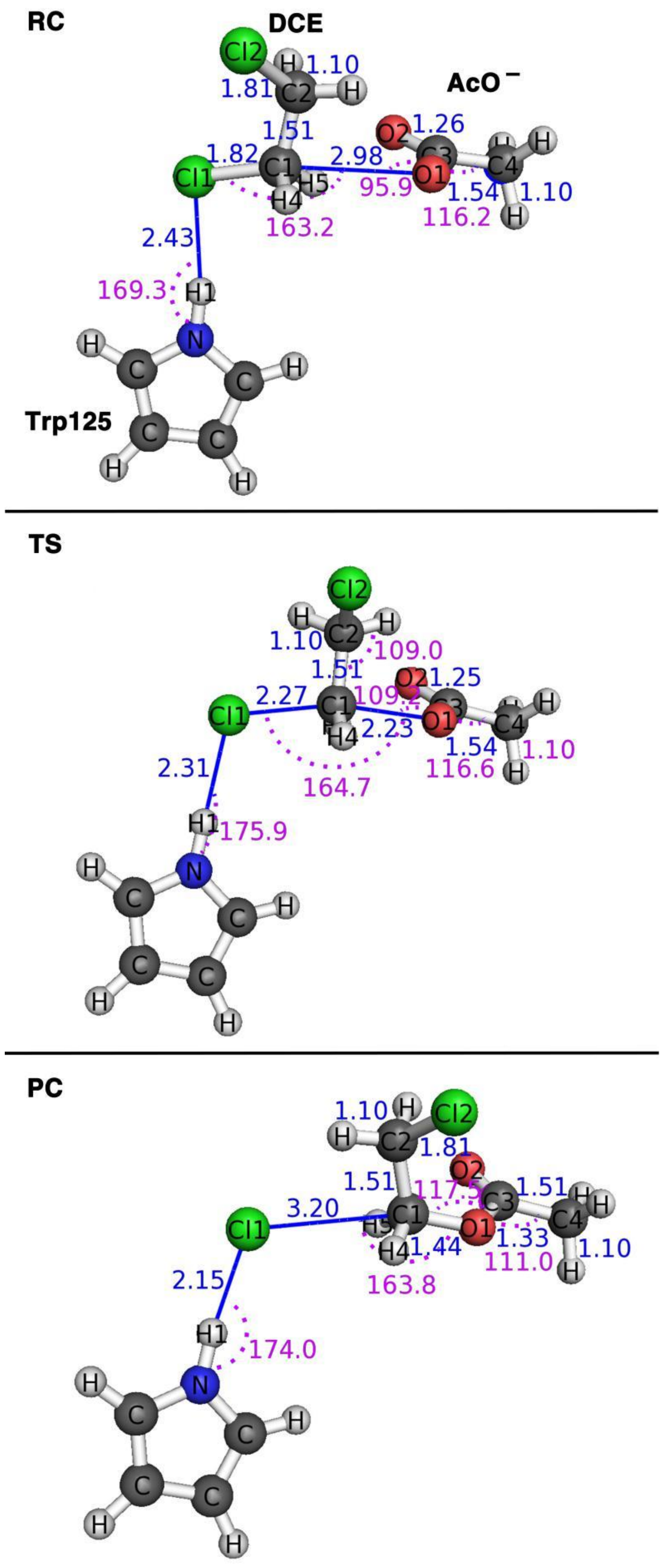

Figure S3. The three stationary points (reactant complex(RC) , transition state(TS), and product complex(RC)) of the AcO^{-} + DCE reaction with one residue (Trp125).

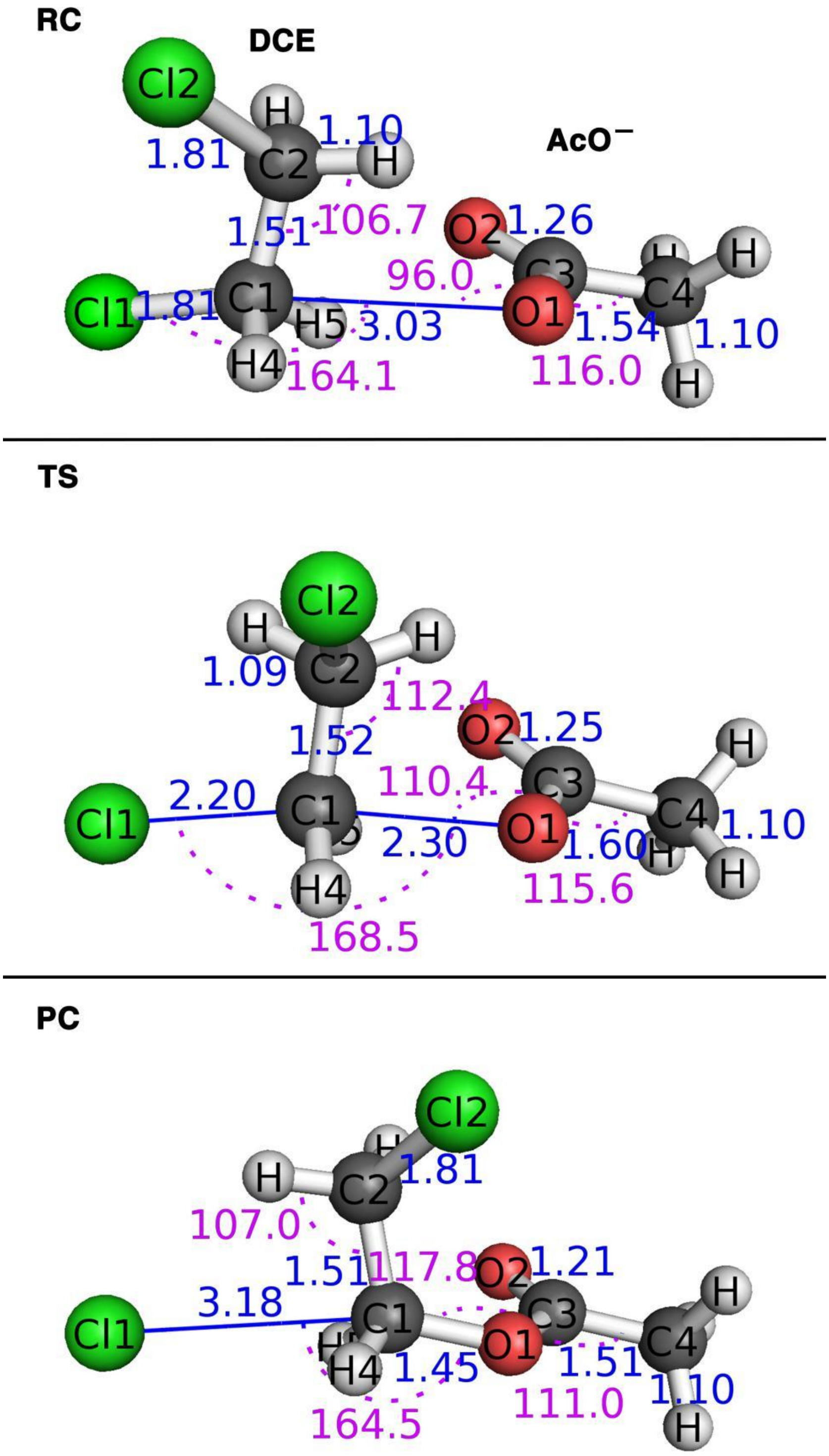

Figure S4. The three stationary points (reactant complex(RC), transition state(TS), and product complex(RC)) of the AcO^{-} + DCE reaction with zero residue.

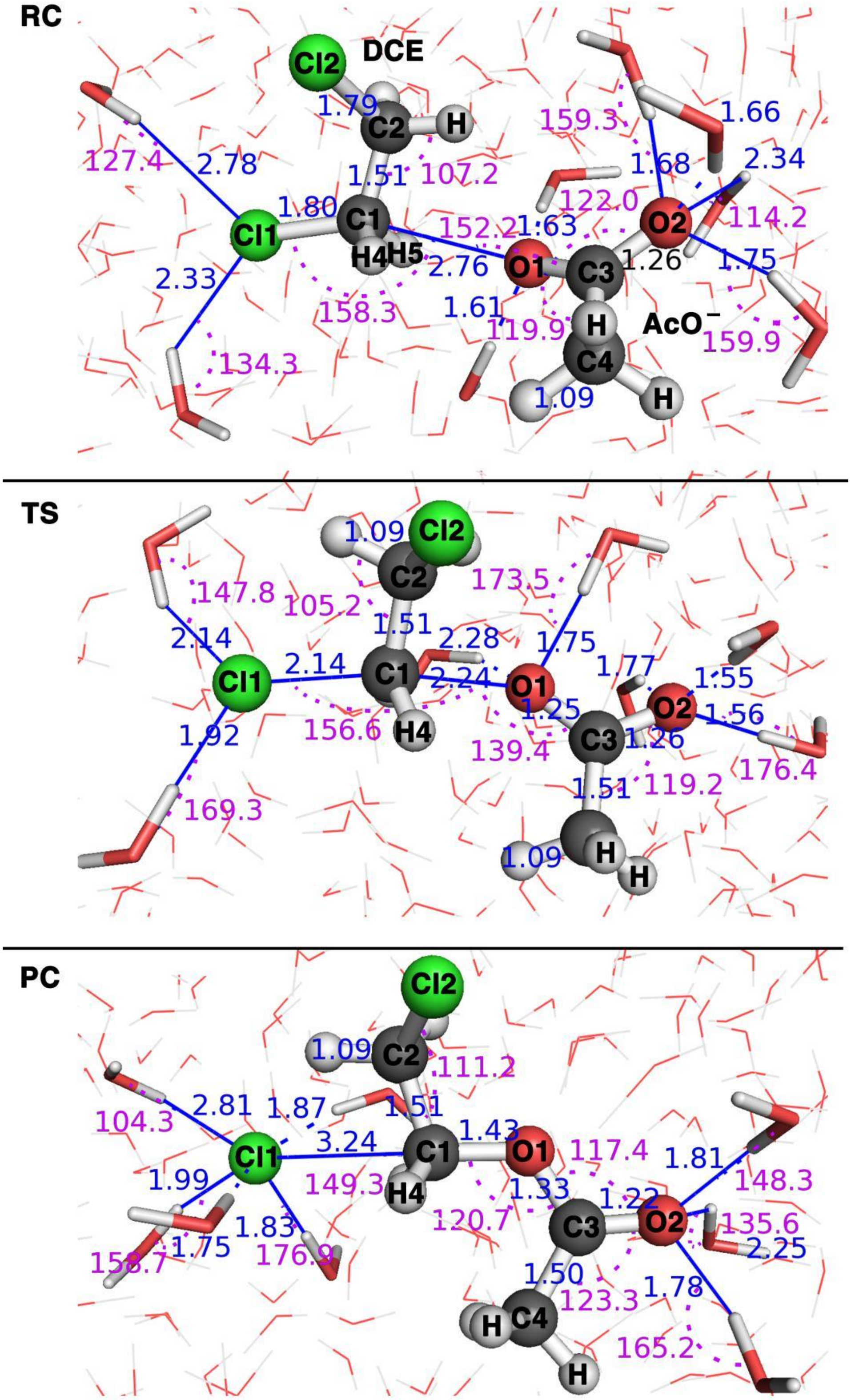

Figure S5. The three stationary points (reactant complex(RC), transition state(TS), and product complex(RC)) of the AcO^{-} + DCE reaction in water.
